# Implementation of an Animal Sporotrichosis Surveillance and Control Program, Southeastern Brazil

**DOI:** 10.3201/eid2703.202863

**Published:** 2021-03

**Authors:** Simone M. Moreira, Elisa H.P. Andrade, Marcelo T. Paiva, Hassan M. Zibaoui, Lauranne A. Salvato, Maria I. Azevedo, Camila S.F. Oliveira, Danielle F.M. Soares, Kelly M. Keller, Sérgio L. Magalhães, Maria H.F. Morais, José R.R. Costa, Camila V. Bastos

**Affiliations:** Instituto Federal de Minas Gerais, Bambuí, Brazil (S.M. Moreira);; Universidade Federal de Minas Gerais, Belo Horizonte, Brazil (E.H.P. Andrade, M.T. Paiva, L.A. Salvato, M.I. Azevedo, C.S.F. Oliveira, D.F.M. Soares, K.M. Keller, C.V. Bastos);; Prefeitura Municipal de Contagem, Contagem, Brazil (H.M. Zibaoui, S.L. Magalhães, M.H.F. Morais, J.R.R. Costa)

**Keywords:** Sporothrix sp., fungi, sporotrichosis, animal surveillance and control program, cats, public health, zoonoses, Brazil

## Abstract

We report the implementation of an animal sporotrichosis surveillance and control program that evaluates strategies to identify suspected and infected cats in a municipality in southeastern Brazil. All adopted measures reinforced the program, although strategies had different abilities to detect the presence of infection.

Sporotrichosis, which is caused by the dimorphic fungi of the genus *Sporothrix*, affects several species of mammal. It often occurs in urban areas that have epidemic conditions (*1*). *Sporothrix brasiliensis* is an emerging species prevalent in Brazil (*2*).

Cats show peculiar signs of the disease and enable the agent to multiply, which favors transmission. Brazil has the highest incidence of sporotrichosis in Latin America because of hyperendemic human sporotrichosis, which is transmitted by cats, particularly in the state of Rio de Janeiro (*3*,*4*), in the southeastern region.

Since sporotrichosis is a zoonosis, in the absence of a specific program, the implementation of a surveillance system in animals that use public services can contribute to the identification of the disease and to the recommendation of practices related to control in municipalities. Thus, data produced are transformed into information and are a triggering factor of the information-decision-action triad, which fuels surveillance and constitutes a decision-making instrument (*5*).

Because of the need to identify infected animals, an animal sporotrichosis surveillance and control program was implemented in a municipality of Minas Gerais state, Brazil. The objective of this study was to evaluate different measures and strategies for identification of cases as part of a feline sporotrichosis surveillance system in this municipality during 2017–2018.

## The Study

We conducted the study in Contagem, a municipality located in the southeastern region of Brazil, in Minas Gerais, which borders Rio de Janeiro state. Contagem has an area of 194,746 km^2^ and ≈663,855 inhabitants (*6*). During 2018, the feline population of this city was estimated to be 8,842 (Zoonosis Department, Health Department of Contagem, Minas Gerais, Brazil, 2019, unpub. data).

The cross-sectional observational analysis encompassed a convenience sample of 165 felines who had cutaneous lesions suggestive of sporotrichosis and resided in the municipality during May 2017–December 2018. Using different strategies, public service veterinarians, and endemic control agents (ECA) (Table), we identified animals that had cutaneous lesions suggestive of sporotrichosis. ECA are public health professionals who work in predefined territories, visiting properties regularly for the control of arboviruses. They were trained by veterinarians to pinpoint animals with lesions suggestive of sporotrichosis.

**Table:** Strategies of identifying suspected sporotrichosis cases by health surveillance professionals in Contagem, Minas Gerais, Brazil, May 2017–December 2018*

Category	Classification of identification strategy	Description
1	Euthanasia at CZC	Cats that had serious injuries suggestive of sporotrichosis were brought by owners for veterinary evaluation at CZC and euthanized because of illness or lack of financial resources for treatment. Sample collection for diagnosis confirmation was conducted at CZC after euthanasia.
2	Active surveillance	Cats that had suspicious skin lesions were identified by endemic control agents during routine home visits for control of arboviruses. Sample collection for diagnosis confirmation was conducted in households.
3	Passive surveillance	Cats with suspicious skin injuries were brought by owners to CZC for veterinary evaluation. Sample collection for diagnosis confirmation was conducted at CZC.
4	Public desexing service	Cats with suspicious skin injuries were identified during a clinical examination before the public desexing service at CZC requested by owners. Sample collection for diagnosis confirmation was conducted at CZC before desexing.

*CZC, Center of Zoonosis Control.

We collected samples in households and in the Center of Zoonosis Control (CZC) in Contagem, a public animal unit in which desexing services and programs for zoonosis control are provided. This study was approved by the Committee for Ethics in Research of the Federal University of Minas Gerais (no. CAAE05012918.4.0000.5149), and use of data was approved by the Health Department of Contagem.

Characterization of cases positive for sporotrichosis was performed by collecting exudates from skin lesions of all suspected cases on sterile swab specimens that were placed in Stuart medium and sent to the Laboratory of Mycology and Mycotoxins of the School of Veterinary of the Federal University of Minas Gerais. Samples were cultivated on Sabouraud agar (KASVI, https://www.kasvi.com.br) containing chloramphenicol and cycloheximide and incubated at 25°C for growth of colonies for up to 10 days. Macroscopic and microscopic characteristics of the colonies were observed to identify fungi of the genus *Sporothrix*. Isolates were identified by PCR based on calmodulin gene sequences (*7*).

**Figure 1:**
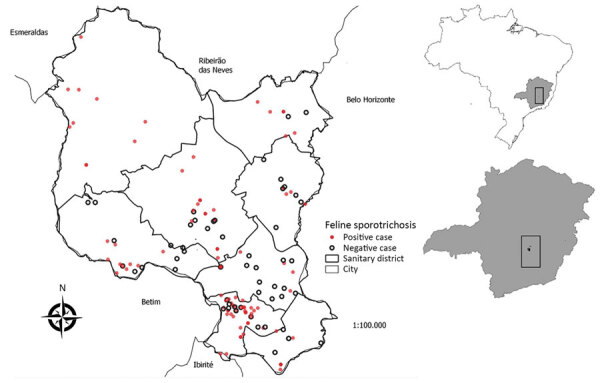
Spatial distribution of cats with cutaneous lesions suggestive of sporotrichosis, according to laboratory diagnosis, Contagem, Minas Gerais, Brazil, May 2017–December 2018. Map was created using QGIS version 3.10 software (https://qgis.org) and the database for Contagem. Inset maps show location of Contagem and Minas Gerais state within Brazil.

Of 165 cats that had cutaneous lesions suggestive of sporotrichosis, which represented 1.86% of the city´s estimated feline population, 103 (62.4%) were considered positive. All health districts (administrative division) had positive cases, demonstrating the spread of the disease throughout the studied territory (Figure 1). Among the positive samples sent for molecular identification (34.3%), all were confirmed as containing *S. brasiliensis*, a species often found in different regions of Brazil (*2*,*7*,*8*) and in parts of Latin America (*9*,*10*). The percentage of positive animals in this was higher than that seen in other locations in Brazil that had epidemics (*1*,*11*).

**Figure 2:**
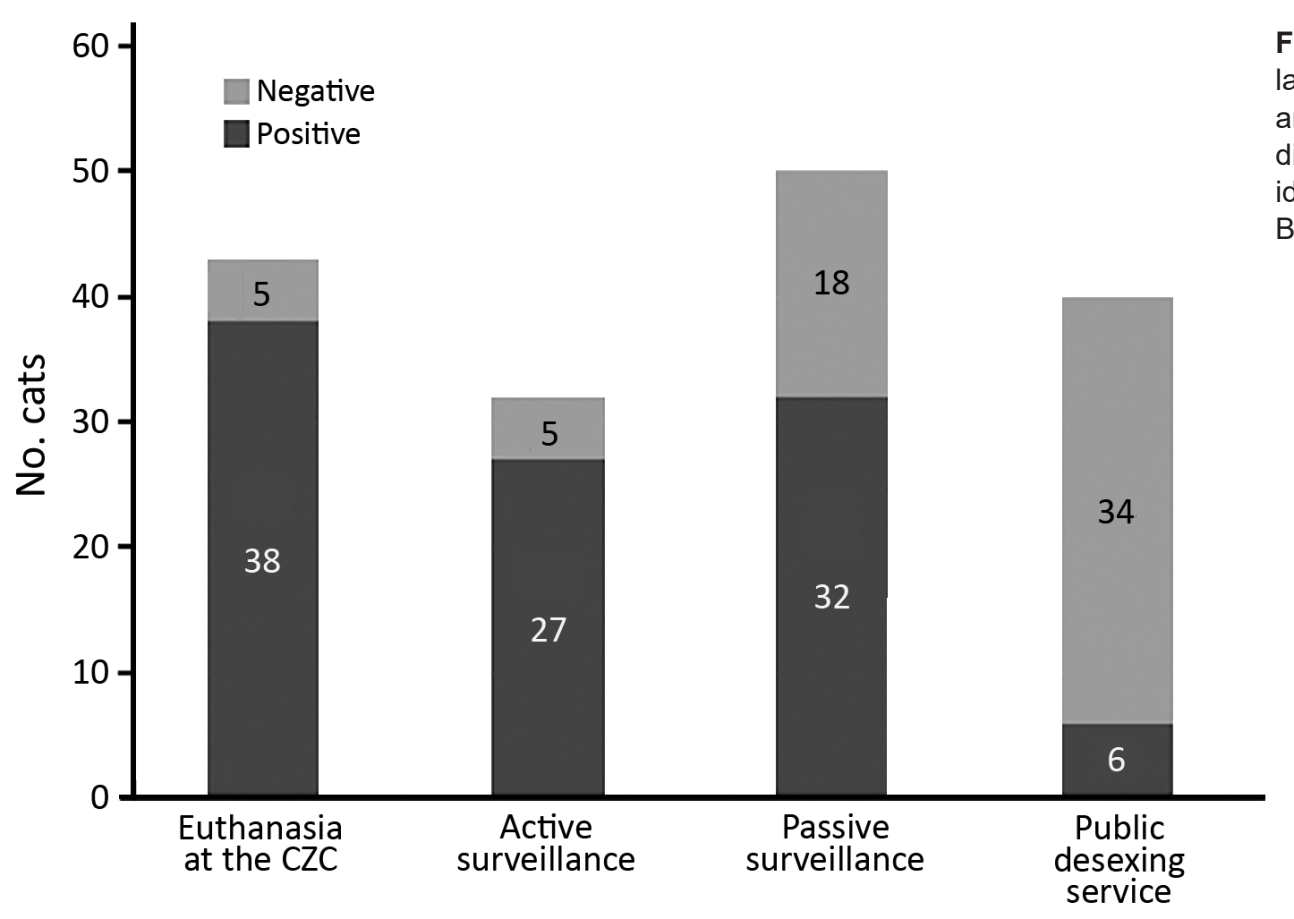
Proportion of cats laboratory-positive for sporotrichosis among animals with suspected disease, according to the strategy of identification, Contagem, Minas Gerais, Brazil, May 2017–December 2018.

The euthanasia service provided 36.9% (38/103) of positive cats. Passive surveillance provide 31.0% (32/103), active surveillance provided 26.2% (27/103) and the public desexing service provided 5.8% (6/103). The ability to detect the infection among the total number of suspected animals identified by each strategy varied (Figure 2). Active surveillance proved to be efficient for identification of infected cats because 84.4% (27/32) had cutaneous lesions suggestive of sporotrichosis obtained by this approach were laboratory positive. In this category, the role of ECA stands out because this group was able to identify animals that had suggestive lesions. This finding demonstrates that awareness and training of these professionals are essential for the early detection of cases in a surveillance program in the cities affected by this zoonosis.

During the study, 50 animals that had suggestive cutaneous lesions were taken to the CZC by inhabitants of Contagem. This action was characterized as passive surveillance. Of these 50 cats, 32 (64.0%) had laboratory confirmed infections. This finding might reflect the awareness of the program among the population, which was promoted mainly by ECA.

Of the 40 cats identified that had lesions suspected of being sporotrichosis by the public desexing service, 6 (15%) were laboratory positive. Among the strategies for obtaining cats suspected of having sporotrichosis, this strategy identified the lowest percentage of positive animals. However, this practice represents an additional form of passive surveillance, which is relevant because of the potential for early identification of the disease resulting from felines submitted for neutering that are clinically evaluated before the surgical procedure.

Conversely, the category of cats that had cutaneous lesions suggestive of sporotrichosis referred for euthanasia at CZC showed the highest positivity (38/43, 88.4%). Animals in this condition were in an advanced stage of the disease. However, the diagnosis made at the time of euthanasia is ineffective in blocking transmission of sporotrichosis because it is most likely that until they died, these animals disseminated the etiologic agent.

## Conclusions

Identifying cats that had cutaneous lesions suggestive of sporotrichosis or that were identified as positive for sporotrichosis contributes to reduction of inappropriate disposal of corpses, which in Contagem are incinerated by an outsourced company. This measure helped reduce environmental contamination and should be part of an animal sporotrichosis control program. Decomposing abandoned dead cats or cats being buried in the domestic environment could represent a risk for increased fungal load in the soil (*12*).

If one considers the health program implemented in the municipality, access to free diagnosis associated with multiple strategies for identifying infected cats (active surveillance, passive surveillance, public desexing service, and euthanasia service) proved to be a useful tool for surveillance and control of feline sporotrichosis. It was possible to identify the disease distributed throughout the territory, and surveillance strategies can, if maintained continuously in the service routine, offer a better situational diagnosis of the disease over time. In our view, the current challenges to the sporotrichosis detection system consist of enhancing training programs and awareness of public health professionals regarding early diagnosis.

We showed relevant advances in the surveillance of feline sporotrichosis. This program is a useful instrument to ensure standardization and efficiency of field actions. Active and passive surveillance were necessary means for initial actions related to completion of the implemented program. Training public health professionals, providing free laboratory diagnosis for animals, and disposing of positive corpses appropriately represent the minimum requirements for support of surveillance and control in municipalities that have their first sporotrichosis cases.
